# Anatomic Locking Plate Fixation for Scaphoid Nonunion

**DOI:** 10.1155/2016/7374101

**Published:** 2016-06-06

**Authors:** Joshua Mirrer, Just Yeung, Anthony Sapienza

**Affiliations:** ^1^NYU Langone Medical Center, New York, NY 10016, USA; ^2^Justin Yeung Plastic Surgery, Calgary, AB, Canada T3A 2N1

## Abstract

Nonunion can occur relatively frequently after scaphoid fracture and appears to be associated with severity of injury. There have been a number of techniques described for bone grafting with or without screw fixation to facilitate fracture healing. However, even with operative fixation of scaphoid fractures with bone grafting nonunion or malunion rates of 5 to 10 percent are still reported. This is the first report of an anatomic locking plate for scaphoid fracture repair in a 25-year-old right hand dominant healthy male.

## 1. Introduction

Nonunion occurs in approximately 5 to 25 percent of scaphoid fractures [1, 2] and appears to be correlated with severity of injury, with displaced fractures displaying higher rates than nondisplaced ones [3, 4]. Additionally, distal pole oblique fractures and proximal pole fractures are considered unstable and are at increased risk of nonunion, malunion, or avascular necrosis [4]. This is especially true for proximal scaphoid fractures due to retrograde blood supply. Despite various modalities of treatment this injury continues to present problems for patient and surgeon alike. There have been a number of techniques described for bone grafting [5, 6] with or without headless screw fixation to facilitate fracture healing. However, even with operative fixation of scaphoid fractures with bone grafting nonunion or malunion rates of 5 to 10 percent are still reported within the literature [1, 7]. This paper describes a novel technique using locking plate and screw fixation of iliac crest bone graft in the treatment of scaphoid nonunion. To our knowledge, this is the first published case of scaphoid fixation using an anatomic locking plate.

## 2. Case Report

**Figure 1:**
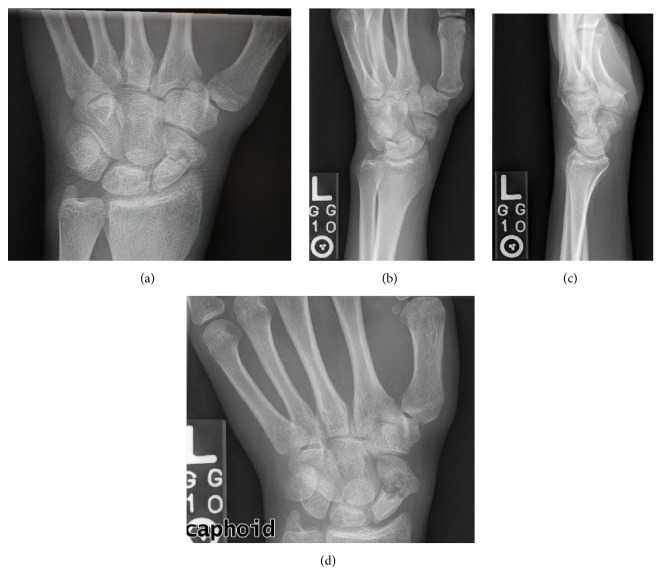


A 25-year-old healthy right hand dominant male who works as a seaman presented to our multidisciplinary hand clinic with persistent radial sided wrist pain. He reports sustaining an injury to his wrist one month prior to presentation from a fall on an outstretched hand. He had been placed in a short arm cast by an outside hospital for immobilization of his left scaphoid waist fracture and was referred to our hand clinic for definitive management. On examination, he had persistent anatomic snuffbox tenderness. Radiographs confirmed a displaced and malrotated fracture pattern (Figures 1(a)–1(d)) with suspicion of acute on chronic injury.

Under general anesthetic, a volar approach to the scaphoid was used to expose the fracture. Direct intraoperative visualization of the fracture site demonstrated central comminution with no signs of healing. There were significant cystic and fibrous changes at the fracture site with sclerotic changes at the proximal pole consistent with fracture nonunion. The nonunion site constituted the central 1/3 of the length of the scaphoid. The fracture nonunion site was debrided until punctuate bleeding was visualized in both proximal and distal poles. This debridement was performed using a combination of curette and burr with copious saline irrigation to minimize the chance of thermal necrosis. Upon completion of debridement it was noted that there were longitudinal fracture lines in the proximal scaphoid pole, as well as a significant bony void secondary to the significant fibrous and sclerotic tissue. Ultimately this was concerning for the potential for propagation of these fracture lines if a headless compression screw was used for fixation.

**Figure 2:**
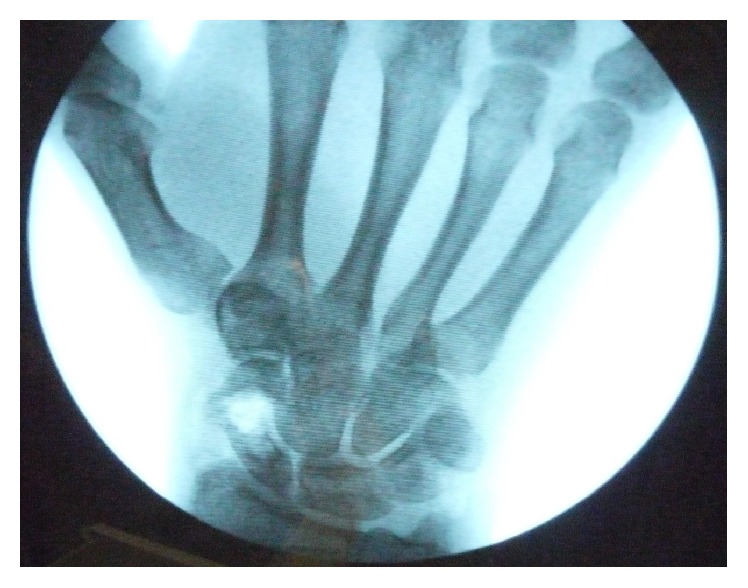


**Figure 3:**
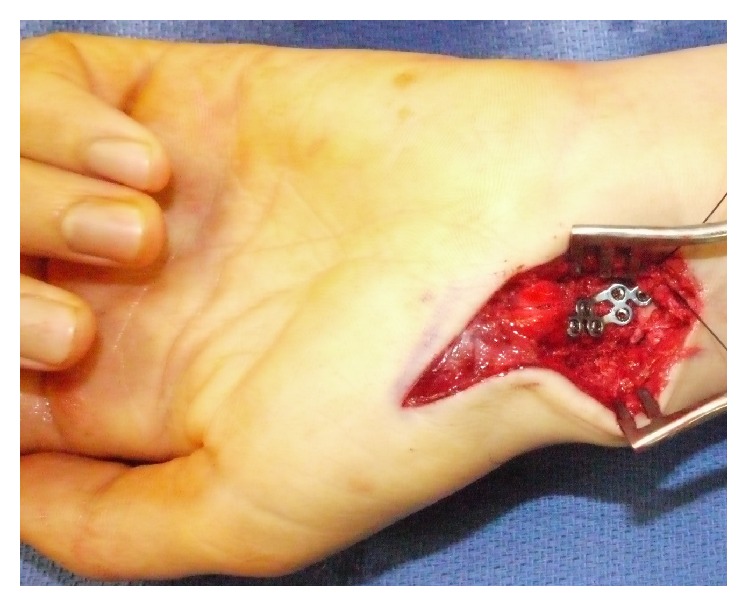


After debridement of the proximal and distal ends, a corticocancellous iliac crest autograft bone graft strut was harvested using a standard anterior approach to the pelvic brim with the incision centered 2 cm inferior and 2 cm posterior to the anterior superior iliac spine. Additional cancellous bone was harvested from the iliac crest to further fill the scaphoid nonunion defect. The corticocancellous bone graft strut was contoured and wedged centrally into the proximal and distal poles of the scaphoid to recreate the appropriate anatomic length of the scaphoid as well as reduce the humpback deformity. Although the bone graft strut was contoured as best as possible, there was still toggle of the proximal and distal poles and the bone graft demonstrated a propensity to extrude out and cause malalignment. The additional cancellous iliac crest bone graft was densely packed around the cortical strut for further stability. A well contoured corticocancellous strut is the best option for scaphoid nonunion. However, this fracture pattern created a unique situation secondary to the longitudinal fracture fragment as well as the large gap of bone which destabilized the proximal and distal segments of the scaphoid. It was determined that plate fixation would best serve to further stabilize the nonunion and buttress of the bone graft to minimize graft extrusion and malalignment. A precontoured Medartis® TriLock 1.5 mm 6-hole scaphoid plate was selected and temporized in position using 0.028 Kirschner wires. Utilizing the variable angle locking system, three screws could be secured into the proximal pole and three screws were secured into the distal pole. Fluoroscopy confirmed appropriate reduction and hardware placement (Figure 2). Gentle wrist range of motion demonstrated excellent stability of the construct with no signs of impingement at the radiocarpal joint (Figure 3).

**Figure 4:**
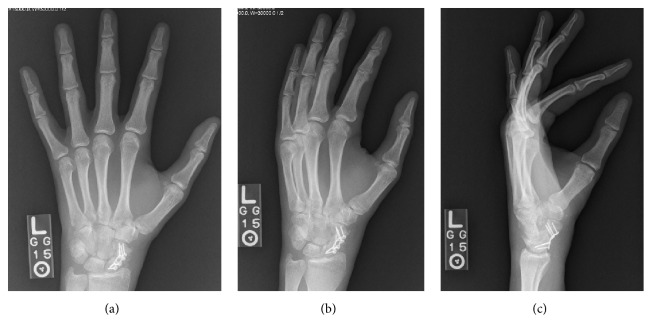


**Figure 5:**
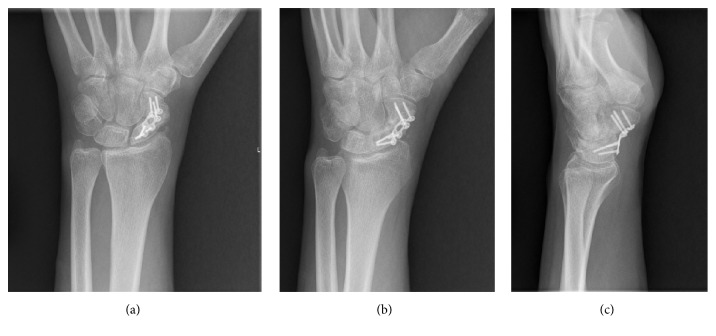


The patient was placed into a Muenster cast for 4 weeks. At that time X-rays were performed out of cast. He was subsequently placed back into a Muenster cast for an additional 4 weeks. At 8 weeks post-op immobilization was discontinued and a radiographic imaging was performed to evaluate bridging trabeculae at the fracture site. The X-rays demonstrated partial uniting of the fracture and we continued the patient immobilization again in a Muenster cast for 4 more weeks (Figures 4(a)–4(c)). Repeat imaging at 12 weeks post-op displayed evidence of bony consolidation with small bridging callus and bony incorporation along the fracture with incomplete graft incorporation (Figures 5(a)–5(c)). He demonstrated excellent mobility, achieving 85% of his contralateral range of motion, with discomfort only at extremes of flexion and extension.

## 3. Discussion

Healing of the scaphoid can sometimes be frustrating for hand surgeons and patients alike. This is often due to the slow healing time, which requires prolonged periods of immobilization. Additionally there is the potential for nonunion, malunion, residual wrist stiffness, and posttraumatic arthritis. Surgical fixation of displaced acute fractures and fracture nonunion remains the standard of care. There are a variety of bone grafting techniques and hardware utilized for stabilization. Although headless compression screws are most commonly used, Kirschner wire fixation and plate fixation have also been described. An in-depth systematic review by Munk and Larsen [15] capturing seventy-five years of data illustrates the variety of techniques available in the armamentarium for treating scaphoid nonunion. However, the authors conclude that because there are no prospective randomized studies comparing the operative treatments, no definitive conclusions can be drawn about their individual efficacy in bony healing.

To our knowledge, seven series have been published on the use of volar plating of the scaphoid (Table 1). Five were performed over twenty years earlier [10–14]; four used the Ender plating system [11–14]. And all five were previously evaluated in the systematic review by Munk and Larsen [15]. Unfortunately, four of these studies were published in foreign journals without English translations [10, 11, 13, 14], which prevents complete examination of their results. One article, however, describes the Ender plating system in detail [12]. This method provides stability, resistance to rotational shearing, and dynamic compression. Although the Ender plating system these authors utilized is different from a miniplate system, it remains a variation of a theme for treatment of scaphoid nonunion and is believed to hold a specific advantage over headless compression screw fixation. The TriLock 1.5 scaphoid plate (Medartis AG, Basel, Switzerland) is precontoured, low-profile (0.8 mm), titanium plate with variable angle locking (±15°) for each plate hole that is anatomically preshaped, allowing ease of intraoperative use. Leixnering et al. [8] and Ghoneim [9] believe that sometimes compression screw fixation is unable to maintain interfragmentary stability, thereby causing delayed or failed healing. The plate and screw fixation construct has the theoretical benefit of multiple divergent screw fixation to lend stability in multiple vectors including torsional stability. Taking this further, the introduction of a locking plate fixation may yield an even stronger construct based on loading bearing mechanics by combining the advantages of the Medartis scaphoid system and some of the advantages of the Ender plating system. To our knowledge, this is the first published case of scaphoid fixation using an anatomic locking plate.

Appropriately contouring the corticocancellous bone graft strut can be technically demanding in a small carpal bone. Additionally, screw position for the multiple screws utilized in plate fixation may require precise localization with fluoroscopic guidance. However, the locking technology allows for stability even with just unicortical screw fixation and allows for minimal concern for intra-articular screw penetration.

Occasionally the fractured poles of the scaphoid are too small to allow enough bony purchase with one screw, or there is fracture comminution that may iatrogenically propagate due to inappropriate hardware choice. The anatomic nature of the plate and wide spread screw fixation can help to reduce rotational instability common with a single headless compression screw as well as allow for variable points of fixation for comminuted fractures. Furthermore, when a significant volume of bone graft material is required, there is risk that the screw can displace the graft or overcompress the fracture site. When plate fixation is utilized in the appropriate clinical setting, it can overcome the drawbacks of a headless compression screw by buttressing the bone graft into position and minimize the potential of overcompression and malangulation of the fracture site. We believe that headless compression screw fixation is still the method of choice for the majority of scaphoid fractures; however there are occasions where an anatomic locking plate may be useful.

Scaphoid plating with a locking anatomic plate can be a useful alternative in cases where a headless compression screw may not be the appropriate fixation method for scaphoid fracture healing.

## Figures and Tables

**Table 1:** Published case series on scaphoid plating.

Author	# of patients	Type of plate	Fracture type	Results
Leixnering et al. (2011) [8]	11	6-hole miniplate nonlocking (Medartis)	Nonunion waist	100% union (median 4 months)
Ghoneim (2011) [9]	14	4-hole straight miniplate nonlocking (Synthes)	Nonunion waist	100% union (median 3.8 months)
Bohler and Ender (1986) [10]	212	Ender blade plate	Nonunion	99% union
Geisl and Pühringer (1986) [11]	37	Ender blade plate	Nonunion	89% union
D. R. Huene and D. S. Huene (1991) [12]	20	Ender blade plate	Nonunion	95% union
Stankovic and Burchhardt (1993) [13]	39	Ender blade plate	Nonunion	95% union
Braun et al. (1993) [14]	16	Plate and screw	Nonunion	94% union
